# Asymmetric Relatedness from Partial Correlation

**DOI:** 10.3390/e24030365

**Published:** 2022-03-03

**Authors:** Carlos Saenz de Pipaon Perez, Andrea Zaccaria, Tiziana Di Matteo

**Affiliations:** 1 Department of Mathematics, King’s College London, The Strand, London WC2R 2LS, UK; cspipaon@gmail.com (C.S.d.P.P.); tiziana.di_matteo@kcl.ac.uk (T.D.M.); 2 Istituto dei Sistemi Complessi (ISC)—CNR, UoS Sapienza, P.le A. Moro, 2, 00185 Rome, Italy; 3 Centro Ricerche Enrico Fermi, Piazza del Viminale, 1, 00184 Rome, Italy; 4 Complexity Science Hub Vienna, Josefstädter Straße 39, A 1080 Vienna, Austria

**Keywords:** complex systems, economic complexity, relatedness, products and services, planar graph, partial correlation

## Abstract

Relatedness is a key concept in economic complexity, since the assessment of the similarity between industrial sectors enables policymakers to design optimal development strategies. However, among the different ways to quantify relatedness, a measure that takes explicitly into account the time correlation structure of exports is still lacking. In this paper, we introduce an asymmetric definition of relatedness by using statistically significant partial correlations between the exports of economic sectors and we apply it to a recently introduced database that integrates the export of physical goods with the export of services. Our asymmetric relatedness is obtained by generalising a recently introduced correlation-filtering algorithm, the partial correlation planar graph, in order to allow its application on multi-sample and multi-variate datasets, and in particular, bipartite temporal networks. The result is a network of economic activities whose links represent the respective influence in terms of temporal correlations; we also compute the statistical confidence of the edges in the network via an adapted bootstrapping procedure. We find that the underlying influence structure of the system leads to the formation of intuitively-related clusters of economic sectors in the network, and to a relatively strong assortative mixing of sectors according to their complexity. Moreover, hub nodes tend to form more robust connections than those in the periphery.

## 1. Introduction

In the past few years, the use of bipartite networks for the representation of real-world complex systems has become widespread in a variety of fields and applications. These networks are usually constructed using multi-sample, multi-variate structured data used to model complex systems such as biological networks (enzymes and reactions [1], genes and diseases [2], plants and pollinators [3]), movies and actors [4,5], authors and papers [5,6], board of directors members and companies [7,8], companies and technologies they patent [9], members of peer-to-peer networks and data provided [10], international NGO branches and cities hosting them [11], supreme court judges and their votes [12], and legislators and bills they sponsor [13].

A prominent example is the bipartite network formed by countries and the products they export. This type of data has been used extensively in the field of economic complexity (EC) [14,15] to assess various quantities of interest for the modelling of the economic development of countries. The first one is the competitiveness of countries and the sophistication of products [16,17,18,19,20], and the relatedness between products, countries, or between countries and products [21,22]. With respect to the datasets implemented in the literature up to now, the dataset we use in this paper adds the inclusion of services to the set of tangible products traditionally considered in the EC literature [23,24].

An agreed definition of relatedness still does not exist, despite the vast number of applications of this concept, that ranges from forecasting industrial upgrading [25] to its use as an explanatory variable in a number of different contexts (see [26] and references therein). In most cases, one computes a projection of the bipartite network (e.g., country-product) onto one of the two sets of nodes to obtain a monopartite network (e.g., product-product) [21,22,27]; the relatedness between the nodes of the target layer is given by the weights of the corresponding links. Since the information content of the projected network is always smaller than that in the bipartite network, the choice of the method employed to achieve this is highly non-trivial. The resulting network should be a meaningful representation of the bipartite network for the specific problem being tackled while minimising the information loss due to the projection. There are several methods available in the literature to carry out this task (see [26,28]); however, to the best of our knowledge, no one takes explicitly into account the temporal structure, with the possible exception of the time-delayed co-occurrences approach described in [23,29] which, however, does not take into account the correlation between the different time series involved. This is a key element, since a comprehensive unveiling of the complex interactions between industrial sectors clearly requires a dynamical perspective.

In this paper, we tackle this issue by quantifying the average influence between industrial sectors in terms of partial correlation. To do so we introduce a framework that generalises a network generation method based on correlation-filtering called the partial correlation planar graph (PCPG) algorithm [30] in order to allow for its use with multi-sample multi-variate datasets. Since this methodology is particularly suitable for bipartite networks such as the ones usually studied in EC, we have called our framework biPCPG. The PCPG is an adaptation of the Planar Maximally Filtered Graph (PMFG) [31] which is in turn a further step from the Minimum Spanning Tree (MST) [32]. Fruitfully applied to financial market dynamics [33], these methods are able to capture the heterogeneity of similarities usually found at different scales of correlation in complex systems thanks to them employing a hierarchical clustering approach rather than a thresholding approach. The advantage of the PMFG over the MST is that, due to its relaxed constraints, its output network contains loops and a larger amount of information than the MST by preserving all the hierarchical properties of the MST [31].

The PCPG [30] adapts the PMFG in order to capture asymmetric interactions among variables in the system, thus producing a directed network. The PCPG achieves this by employing an edge-weighting scheme based on partial correlations, which are a measure of how the correlation of two variables is affected by a third variable. More specifically, the so-called *influence* (the difference between correlation and partial correlation) is employed to measure the similarities in the system and is used as a metric to select the edges included in the network. In our case, this formulation of relatedness allows asymmetries to be detected in the system.

As a result, the PCPG network is a weighted, connected, directed network that includes the MST as a subgraph as well as allowing for other substructures such as loops and cliques of three and four elements which add to the information content of the graph [31]. The fact that the links present in the PCPG are mostly those which correspond to the largest correlations in the system ensures the statistical robustness of the network to a high extent [34].

The PCPG was originally developed for its use on multi-variate datasets of only one sample: the time series of different stocks. In our case we have the export time series, so not only many variables (the different products) but also many samples, one of each country. In this paper we propose an extension of the PCPG, that we call biPCPG, to allow its application on multi-sample and multi-variate datasets, e.g., the export time series, by product, of many countries.

Our proposed extension to the PCPG method involves the preparation of the multi-sample dataset in order to apply the PCPG algorithm. This is achieved by structuring the dataset into a set of correlation matrices among the time series of products exported by countries, averaging these, and applying the existing PCPG procedure. Following similar principles, we also adapt an existing bootstrapping procedure (see [34]) in order to determine the statistical reliability of the links present in the resulting network.

The contribution of this paper is many fold. Firstly, the biPCPG framework opens the possibility of the application of the PCPG algorithm to a wide variety of datasets with a multi-sample and multi-variate structure, including, but not limiting to, the ones usually analysed using the EC framework. Furthermore, the data-processing methodology introduced here could be utilised to apply other correlation-filtering algorithms for network generation (e.g., [31,33]).

Secondly, this paper introduces a network which describes the asymmetric relatedness among physical products (manufacturing) and services. This is an addition with respect to the networks usually present in the literature, such as the product space [21] and product taxonomy network [22], which are constituted only by products.

Thirdly, this paper introduces an adapted bootstrapping procedure to asses the reliability of the edges present in a network generated from multi-sample multi-variate datasets. Similarly to the network-generating framework, this bootstrapping procedure can be utilised to asses the reliability of edges in networks generated using alternative correlation-filtering methods with datasets with this structure.

Fourthly, in order to assess the information content of the biPCPG network we calculate two assortativity measures and run a community detection procedure, finding that meaningful clusters and connections emerge, as well as a relevant complexity-related assortativity. In summary, the biPCPG analysis unveils the average influence between industrial and service sectors, efficiently encapsulating the information about the correlation structure of the system.

Finally, we provide a Python package named “biPCPG” [35] with its documentation hosted in [36]. The 0.1.0 version of this package was used to perform all the calculations done in this paper, including the data-handling, biPCPG network generation, bootstrapping procedure and calculations done on the biPCPG network. It is worth noting that the package has a modular structure such that the data-handling and the generation of the biPCPG network are computed independently of each other. This allows the user to, for example, utilise the data-handling module to prepare a multi-sample multi-variate dataset for an alternative correlation-filtering method, or to implement the PCPG algorithm on a dataset of her choice, without the need for the dataset to have a multi-sample multi-variate structure. To the best of our knowledge, the PCPG module in the biPCPG package is the first publicly available Python implementation of the PCPG algorithm.

The rest of this paper is organised as follows. In Section 2, we describe the dataset used in this investigation and the cleaning procedure performed on it. In Section 3, we describe the set of methods to generate the biPCPG network and comment on the resulting network. In the result sections we describe the assortativity calculations and community detection procedure done on the biPCPG network and show the results obtained. Section 5 concludes.

## 2. Data Description and Preprocessing

The dataset used in this research project is an integration of the United Nations Commodity Trade Statistics Database (UN-COMTRADE—https://comtrade.un.org, accessed on 13 February 2019) and the International Monetary Fund’s Balance of Payments data (BPM6) [37], relative to physical goods and service exports respectively. This integrated dataset was introduced in a World Bank working paper [23]. The UN-COMTRADE data consists of the amount of exports from each country per category of products (in USD). The categorisation of products is given by the World Customs Organization’s (WCO) Harmonized System 2007 edition (HS2007) [38], which classifies products by using a hierarchical six-digit code depending on the category of the product. The IMF BPM6 dataset consists of the amount (in USD) of services provided abroad by each country and is collected according to the 6th edition of its manual, provided by the International Monetary Fund (IMF). Henceforth, we will globally refer to the collection of products in COMTRADE and services in BPM6 as *sectors*.

The hierarchical structure of the HS classification allows for an aggregation from the most granular six-digit level, consisting in about 5000 different products, into a coarser two-digit level. A further aggregation of a few small (in terms of export quantities) two-digit sectors into a single two-digit sector was also performed in this dataset, leaving a total of 78, roughly homogeneous aggregated product sectors at the two-digit level. From the BPM6 part of the dataset, there are a further 22 service sectors at a comparable level of aggregation.

The aggregated dataset used in our study is therefore comprised of 78+22=100 sectors of products and services, these are listed in Table A1 in Appendix D. The data span a total of 22 years, from 1995 to 2016. As there are missing data points in some years for several countries, we apply a sanitation procedure where only countries with complete data for all sectors throughout the 22 years are kept. This reduces the dataset to from 129 countries to 99 countries. The analysed dataset has a total 99×100=9900 time series of length 22, with no missing values, representing the amount of product exports or service provisions in USD for each country.

In order to perform specific calculations (see Section 4.2), the 100 sectors in the dataset must be aggregated one level further. The product sectors can be further aggregated using what the WCO refers to as *sections*. The WCO provides a total of 21 sections which are available at [38]. In this case, services sectors can be aggregated into a single “section”. Thus, in our aggregated dataset we have a total of 22 sections of sectors—21 product sections arising from the HS2007 classification, and one additional section containing the service sectors from the BPM6 dataset.

### Revealed Comparative Advantage Matrices

The raw data used to construct in this paper are the amount of exports $E_{c,p}^{y}$ (in USD) of a sector *p* (product or service) by a country *c* in year *y*. We compute the Revealed Comparative Advantage (RCA) [39] as

(1) $$\begin{array}{cc} RCA_{c,p}^{y} & =\frac{\mathrm{ratio}\;\mathrm{of}\;c’s\;\mathrm{exports}\;\mathrm{of}\;p\;\mathrm{to}\;\mathrm{the}\;\mathrm{total}\;\mathrm{exports}\;\mathrm{of}\;c\;\mathrm{in}\;\mathrm{year}\;y}{\mathrm{ratio}\;\mathrm{of}\;\mathrm{the}\;\mathrm{world}’s\;\mathrm{exports}\;\mathrm{of}\;p\;\mathrm{to}\;\mathrm{the}\;\mathrm{total}\;\mathrm{world}’s\;\mathrm{exports}\;\mathrm{of}\;\mathrm{all}\;\sec\mathrm{tors}\;\mathrm{in}\;\mathrm{year}\;y}\\ \; & =\frac{E_{c,p}^{y}/\sum_{p^{\prime }\in P}E_{c,p^{\prime }}^{y}}{\sum_{c^{\prime }\in C}E_{c^{\prime },p}^{y}/\sum_{c^{\prime }\in C,p^{\prime }\in P}E_{c^{\prime },p^{\prime }}^{y}} \end{array}$$

where *P* and *C* are the sets of unique sectors and unique countries in the dataset discussed above.

The use RCA is ubiquitous in the EC literature, because removes trivial dependencies from the sectors’ and countries’ size. When the $RCA_{c,p}^{y}$ is above 1, the country is said to have a revealed comparative advantage in exporting a given sector in that year. Conversely, when $RCA_{c,p}^{y}$ is below 1 the country can be thought of as not being very competitive in that particular sector. Finally, when $RCA_{c,p}^{y}$ is equal to 1 the country has the expected (average) share of the world’s exports in the given sector and year.

Therefore, the dataset on which we perform the following calculations consists of time series $RCA_{c,p}=\left(RCA_{c,p}^{y}:y\in Y\right)$ for 99 countries and 100 sectors, where *Y* is the index set of years [1995,2016]. The data is then shaped into a set of 22 matrices ${\mathrm{RCA}}^{y}$, one for each year, where each row represents a country, each column represents a sector and each entry is the corresponding $RCA_{c,p}^{y}$ value.

## 3. Methods: The biPCPG Framework

### 3.1. Methodology Description

**Figure 1:**
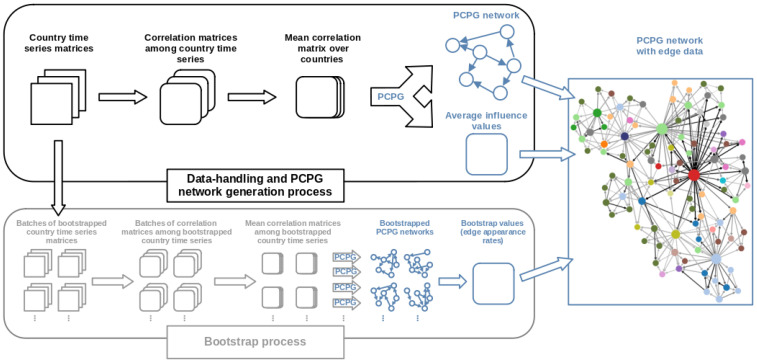
Flowchart of procedures and methods involved in obtaining the final biPCPG network.

Before discussing the detailed implementation of the biPCPG methodology, here we provide a summarised description of our procedure; a visual representation can be found in Figure 1.

Given the multi-sample nature of the dataset analysed, a series of data-preprocessing steps are needed before the application of PCPG. The PCPG algorithm takes a single correlation matrix as an input and outputs a network (see Section 3.5). In order to obtain our biPCPG network, along with reliability values for its edges from a multi-sample dataset, we need two main procedures, a “Network generating procedure” and a “Bootstrapping procedure”.

The “Network generating procedure” is shown in the black box in Figure 1 and deals with the data handling necessary to obtain a PCPG network from a dataset with a multi-sample structure. In our case, we are interested in obtaining a biPCPG network where nodes are sectors, therefore the input matrix should describe the correlations between sectors.

To find this input correlation matrix, the initial step is to shape the dataset such that, for each country, we have a matrix where the columns are the relevant time series of each sector. We then compute a correlation matrix for each of these time series matrices. Finally, we average these correlation matrices over countries to obtain an average correlation matrix which serves as the input to the PCPG algorithm, i.e., the last step in the biPCPG framework. The output of the biPCPG algorithm is the network we refer to as *G*, as well as the weights of the edges in contains, i.e., the average influence between sectors.

The “Bootstrapping procedure” of our framework, shown in the grey box in Figure 1, deals with the bootstrapping procedure necessary to asses the reliability of the edges in the biPCPG network obtained. This starts from the country time series matrices, which are bootstrapped *R* times, obtaining a “batch” of replicates each time. Each of these batches contains *C* matrices, one for each country, where the rows have been drawn coherently from their corresponding original country matrices. This is done in order to randomise the time dimension while preserving the correlation structure across countries (see Section 3.6). We then replicate the “Network generating procedure” described above by treating each batch of replicates as a new dataset of country time series matrices and follow the steps to obtain a replicate biPCPG network. This means that, for each batch, we calculate a correlation matrix for every time series matrix, we then average across these correlation matrices and use the average correlation matrix as an input to the PCPG algorithm. Repeating this procedure for all *R* batches we obtain *R* replicate networks. We find the fraction of times each edge in *G* appears in the replicate networks, which is a measure of the reliability of the edge.

### 3.2. Partial Correlations and Average Influence: Definitions

As described in the original PCPG paper (see [30]), the starting point of our analysis is the *partial correlation*, which measures the effect that a random variable *Z* has on the correlation between two other random variables, *X* and *Y*. The partial correlation ρ(X,Y:Z) is defined in terms of the Pearson correlations ρ(·,·) between the three variables, formally

(2) $$\rho \left(X,Y:Z\right)\;=\;\frac{\rho (X,Y)-\rho (X,Z)\rho (Y,Z)}{\sqrt{\left[1-\rho^{2}\left(X,Z\right)\right]\left[1-\rho^{2}\left(Y,Z\right)\right]}}.$$

A small value of ρ(X,Y:Z) may be ambiguous, as this could be due to the correlations among the three variables being small; or due to variable *Z* having a strong effect on the correlation between *X* and *Y*, which is generally the interesting case. In order to discriminate between these two cases the *correlation influence* or *influence* of variable *Z* on the pair of elements *X* and *Y* is used. This is defined as

(3) d(X,Y:Z)≡ρ(X,Y)−ρ(X,Y:Z).

We define the *average influence* of variable *Z* on the correlations between *X* and all other variables in the system as follows:

(4) $$d\left(X:Z\right)\;=\;{\langle d\left(X,Y:Z\right)\rangle }_{Y\neq X}.$$

We anticipate that the average influence will be the input of the network building algorithm also described in [30].

Note that, potentially, there could be certain values of *measured* correlations ρ(X,Y), ρ(X,Z) and ρ(Y,Z) that lead to a *measured* partial correlation ρ(X,Y:Z), to be out of its defined range [−1,1]. In our analysis, this occurred in 0.02% of the partial correlations computed. In these cases, partial correlations were set to be undefined (*NaN* in programming terms) which in turn makes the influence values based on these partial correlations also undefined. Similarly to the undefined correlation values described above, these undefined influences are not included in calculation of average influence d(X:Z).

Some of the values obtained for ρ(X,Y), ρ(X,Y:Z), d(X,Y:Z) and d(X:Z) in our dataset and their interpretation are discussed in Section 3.4. An important point is that, in general, d(X:Z)≠d(Z:X): the influence is asymmetric, and the largest among these two quantities indicates the main direction of influence between *X* and *Z*. For example, in our dataset when X=Glass and Z=Furniture, the average influence of Furniture on Glass d(X:Z)=0.03 while the corresponding reverse average influence of Glass on Furniture d(Z:X)=0.29, suggesting that the direction of influence is from Glass to Furniture and not vice-versa. This, however, is an example of a clear-cut case, where difference between the two average influence values is not small. In general, these differences tend to be much smaller. This can be an effect of the complex relationship and mutual interaction between the economic sectors, or a consequence of the noise present in the data. This makes a bootstrapping procedure necessary in order to asses the statistical confidence in the overall direction of influence, as well as the average influence values themselves. We will discuss the bootstrapping procedure in Section 3.6.

### 3.3. Average Correlation Matrix

The input to the PCPG algorithm is a correlation matrix [30]. In our procedure, to allow its use on our multi-sample dataset, this correlation matrix is replaced by an average correlation matrix over countries. In order to obtain this average correlation matrix, we reshape the 22 ${\mathrm{RCA}}^{y}$ matrices into a total of C=99 matrices, one for each country, each consisting of T=22 rows and P=100 columns. We denote these ${\mathrm{TS}}_{c}$, c∈1,…,C. In this way, the columns of each matrix ${\mathrm{TS}}_{c}$ are the $RCA_{c,p}$ time series of the given country *c*, where each column represents a sector *p* in the dataset.

In order to obtain the input matrix to the PCPG algorithm, we first find *C* correlation matrices denoted $K_{c}$, c∈1,…,C from the pair correlations between the columns of each matrix ${\mathrm{TS}}_{c}$. Thus the entries of the country correlation matrix $K_{c}$ are given by

(5) $${\left(K_{c}\right)}_{p,p^{\prime }}\;=\;\rho \left({\left(TS_{c}\right)}_{*,p}\;,{\left(TS_{c}\right)}_{*,p^{\prime }}\right)\;=\;\rho \left(RCA_{c,p}\;,RCA_{c,p^{\prime }}\right)$$

where ρ is the Pearson correlation, the subscript ∗,p denotes the column *p* of the matrix and $RCA_{c,p}$ is the RCA time series for country *c* and sector *p*.

For each correlation value we obtain *p*-value via a two-sided T-test procedure [40]. Given we are performing multiple tests, we apply a False Discovery Rate (FDR) correction to obtain *adjusted* *p*-values via the Benjamini–Hochberg (BH) procedure [41]. We choose the BH procedure since it ultimately allows the inclusion of more information in the biPCPG network than a more restrictive correction procedure such as the Bonferroni correction [42]. Note that the FDR correction has been extensively used in the literature for the statistical validation of networks and, in particular, it has been previously used to validate networks representing bipartite complex systems [43].

We reject non-statistically significant correlation samples when the adjusted *p*-value is above a critical value of 0.01. In these cases, the corresponding entries to the $K_{c}$ matrix are marked as undefined. The same procedure for obtaining country correlation matrices was also performed without the FDR correction for the 0.01 and alternative critical values. This produced networks which have the same main features as the network presented below, including the main hub nodes, clusters of sectors and communities detected.

Once the country correlation matrices $K_{c}$ are found, we then compute the element-wise mean of these matrices, obtaining the average correlation matrix $\bar{K}$ with entries

(6) $${\bar{K}}_{p,p^{\prime }}=\frac{1}{C}\sum_{c=1}^{C}{\left(K_{c}\right)}_{p,p^{\prime }}\;,$$

where row and column indices *p* and $p^{\prime }$ denote economic sectors. Any undefined correlation is discarded during the averaging process.

Note that, using this notation, the correlations ρ(·,·) mentioned in Section 3.2, are replaced by the average correlations ${\bar{K}}_{p,p^{\prime }}$ described here. This leads to an equivalent expression for the partial correlation

(7) $$\rho \left(p,p^{\prime }:p^{\prime \prime }\right)\;=\;\frac{{\bar{K}}_{p,p^{\prime }}-{\bar{K}}_{p,p^{\prime \prime }}{\bar{K}}_{p^{\prime },p^{\prime \prime }}}{\sqrt{\left[1-{\left({\bar{K}}_{p,p^{\prime \prime }}\right)}^{2}\right]\left[1-{\left({\bar{K}}_{p^{\prime },p^{\prime \prime }}\right)}^{2}\right]}}.$$

### 3.4. Partial Correlation and Average Influence: Empirical Analysis

In order to clarify the meaning of the intermediate quantities that are used to build the biPCPG network, we devote this subsection to the discussion of some empirical features.

Bearing in mind how the influence of a variable on the correlation of two other variables is defined (see Equation (3)), we explore four examples of the results obtained from these computations. Note that, in the description below, the variables *X*, *Y* and *Z* used in the definition of Equation (3), are replaced by sectors of our system. Thus, the partial correlation column in Table 1 describes the average correlation, ${\bar{K}}_{p,p^{\prime }}$, between sectors *p* and $p^{\prime }$ accounting for the effect of a third sector $p^{\prime \prime }$, and similarly for the influence column. We therefore denote these quantities $\rho (p,p^{\prime }:p^{\prime \prime })$ and $d(p,p^{\prime }:p^{\prime \prime })$, respectively.

Example 1 shown in Table 1 is an example of the case described in Section 3.2, which shows a very small partial correlation due to all correlations among the three variables being small. By definition, this makes the resultant influence value is small, which reduces the average influence of the sector “Other textile” on the sector “Cereals”, making the appearance of this edge in the network less probable.

Example 2 also shows a case where the partial correlation between *p* and $p^{\prime }$, accounting for the effect of $p^{\prime \prime }$, is small. However, contrary to the case in Example 1, this is due to $p^{\prime \prime }$ strongly affecting the correlation between *p* and $p^{\prime }$, i.e., $\rho \left(p,p^{\prime }\right)∼\rho \left(p,p^{\prime \prime }\right)\rho \left(p^{\prime },p^{\prime \prime }\right)$. Therefore, the resulting influence is relatively high, which increases the probability of an edge from “Cultural” to “Audiovisual” being present in the biPCPG network. In addition, note that the probability of an edge from “Cultural” to “Audiovisual” also increases under these results, due to the symmetry between the *p* and $p^{\prime }$ variables.

In Example 3, we have a case where the correlation between *p* and $p^{\prime }$ is relatively strong and variable $p^{\prime \prime }$ has a small effect on it. This is due to the similar values of the correlation $\rho (p,p^{\prime })$ and the partial correlation $\rho (p,p^{\prime }:p^{\prime \prime })$. Therefore, the resulting influence of “Knitted clothing” on the correlation between the “Pigments” and “Aluminium” sectors is close to zero.

Finally, Example 4 shows a seemingly counter-intuitive case where the correlation between *p* and $p^{\prime }$ is small while their partial correlation given $p^{\prime \prime }$ is negative, yielding a high influence. A negative partial correlation occurs when the correlation between *p* and $p^{\prime }$ is small but both *p* and $p^{\prime }$ have a high correlation with $p^{\prime \prime }$. In this case, the influence of “Plastics” can be interpreted as preventing the correlation $\rho (p,p^{\prime })$ between “Vehicles” and “Earths and stone” from being lower, or being negative.

**Figure 2:**
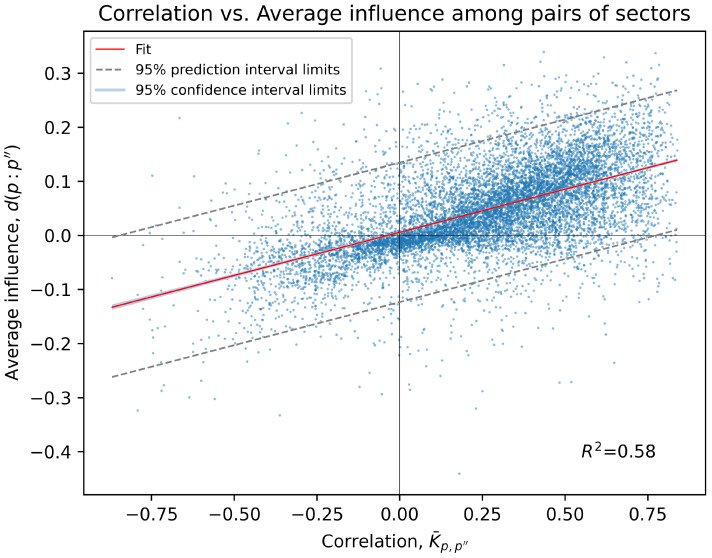
Plot showing correlation and average influence values among all 9900 pairs of sectors in the system. A line of best fit among the points is shown in red along with the coefficient of determination $R^{2}=0.58$, with the 95% confidence interval limits in light blue and the 95% prediction interval limits in dashed grey lines. Note the confidence interval is so narrow it is only visible at the edges of the red best fit line upon close inspection.

It is important to note that the average influence values among sector pairs determine the structure of any PCPG network (see Section 3.5). Figure 2 displays a scatter plot that shows the correlation ρ(·,·) and average influence d(·,·) among all N(N−1)=9900 pairs of sectors in our biPCPG network. Note that this includes data points for both $d(p:p^{\prime \prime })$ and $d(p^{\prime \prime }:p)$ influences at the same horizontal coordinate as the correlation between *p* and $p^{\prime \prime }$ is symmetric.

This plot shows that the average influence between a pair of sectors is highly correlated with the correlation between the same pair of sectors, showing a very narrow 95% confidence interval (barely visible as it is only slightly wider than the fit line). See Appendix B for details on the calculation of the confidence and prediction intervals shown in Figure 2.

This is not surprising given how the average influence is calculated; however, the relatively high coefficient of determination $R^{2}=0.58$ indicates that, generally, the partial correlation values obtained are relatively small. This may be due to there actually not being large influences between the sectors, or due to limitations of the dataset. For example, hidden influences between the sectors could potentially be detected in datasets with longer time series.

In Figure 2, we can observe that most of the correlations (around 80%) are positive. Around 10.7% of the pairs of sectors with positive correlations have an average influence below zero. This quantity is over an order of magnitude larger than its counterpart, the percentage of pairs of sectors with negative correlation but a positive average influence, which is around 0.47%.

### 3.5. Network Construction

The construction algorithm of a PCPG network starts with a list of the N(N−1) average influence values in decreasing order and an empty graph of *N* nodes and no edges, where *N* is the number of variables in the system. In our case, we have N=100 economic sectors. We then cycle through the sorted list, starting with the largest average influence value found, e.g., $d(p:p^{\prime \prime })$, where *p* and $p^{\prime \prime }$ are a given pair of products. The edge $p^{\prime \prime }\to p$ is included in the network if and only if the resulting network is still planar and the edge $p\to p^{\prime \prime }$ has not been included already. We stop adding edges if adding the next edge in the list would break the planarity of the graph. This procedure ensures two things: (i) only the largest among $d(p:p^{\prime \prime })$ and $d(p^{\prime \prime }:p)$ will be included in the network, and (ii) the final network has 3(N−2) edges. It is important to note that for a given input correlation matrix of size N×N the PCPG network will always have 3(N−2) edges and that the identity of these edges solely depends on the correlation values in the input matrix.

The final result of this procedure is what we refer to as the biPCPG network, *G*. Naturally, we also obtain the average influence *d* associated to each edge in *G*, as well as the network’s adjacency matrix A defined as

(8) $$A_{p,p^{\prime \prime }}\;=\;\left\{\begin{array}{cc} 1 & \mathrm{if}\;\mathrm{edge}\;p\to p^{\prime \prime }\in G,\\ 0 & \mathrm{otherwise}. \end{array}\right.$$

### 3.6. biPCPG Bootstrapping

To assess the reliability of the links in the biPCPG network, we adapt a bootstrapping procedure originally introduced in [34]. The aim is to obtain a bootstrap value for each link which is proportional to the reliability of the link.

We build *R* batches, where the matrices to be bootstrapped in each batch are the time series matrices of all countries ${\mathrm{TS}}_{c}\;\forall \;c\in 1,\ldots ,C$. From each matrix ${\mathrm{TS}}_{c}$, a replicate time series matrix ${\mathrm{TS}}_{c}^{r}\;\forall \;r\in 1,\ldots ,R$ is obtained, where *R* = 1000 is the total number of batches. An important feature of our procedure is how the null model, i.e., the replicate time series matrices, is generated. For each batch, the bootstrapping of the time series matrices is done coherently across countries. This means rows are drawn with repetition from each of the country matrices *jointly*—the same row indices are selected across the matrices. In addition, the new locations of the selected rows in their corresponding replicate matrices are exactly the same. This way, in the replicate time series matrices, ${\mathrm{TS}}_{c}^{r}$, the time structure of the time series is destroyed while preserving the country-level correlations.

Take, for example, the first batch, r=1. In order to obtain the first batch of replicate matrices ${\mathrm{TS}}_{c}^{1}\;\forall \;c\in 1,\ldots ,C$, we randomly select a sequence of T=22 row indices, allowing repetitions. These row indices denote which rows from the original matrices ${\mathrm{TS}}_{c}$ are included in the corresponding replicates ${\mathrm{TS}}_{c}^{1}$ in this batch, as well as their order. This way, any row of a replicate matrix in this first batch will contain data points corresponding to the same year as rows of the same index in all the other replicate matrices in the batch.

After all the replicate matrices are obtained for all countries and batches, we calculate a replicate correlation matrix $K_{c}^{r}$ for each of them, rejecting non-statistically significant samples as described in Section 3.3. We then find the element-wise mean of the replicate correlation matrices in each batch *r*, obtaining *R* replicate average correlation matrices ${\bar{K}}^{r}$ where

(9) $${\bar{K}}_{p,p^{\prime }}^{r}\;=\;\frac{1}{C}\sum_{c=1}^{C}{\left(K_{c}^{r}\right)}_{p,p^{\prime }}\;.$$

Note that, similarly to the replicate time series matrices, in these replicate correlations matrices the time structure of the time series is destroyed while preserving the country-level correlations due to the way the bootstrapping has been performed.

We then apply the PCPG algorithm described in Section 3.5 to each matrix ${\bar{K}}^{r}$, obtaining *R* replicate adjacency matrices, $A^{r}\;\forall \;r\in 1,\ldots ,R$.

To compute the bootstrap value, $b_{p,p^{\prime \prime }}$, for each link $p\to p^{\prime \prime }$, we evaluate the number of time the link appears in the replicate adjacency matrices $A^{r}$, and normalise by the number of replicates *R*, formally

(10) $$b_{p,p^{\prime \prime }}\;=\;\frac{\sum_{r=1}^{R}A_{p,p^{\prime \prime }}^{r}}{R}$$

Each bootstrap value is therefore some number in the interval [0-1] and is proportional to the reliability of the link.

## 4. Results

### 4.1. Descriptive Analysis of the biPCPG Network

**Figure 3:**
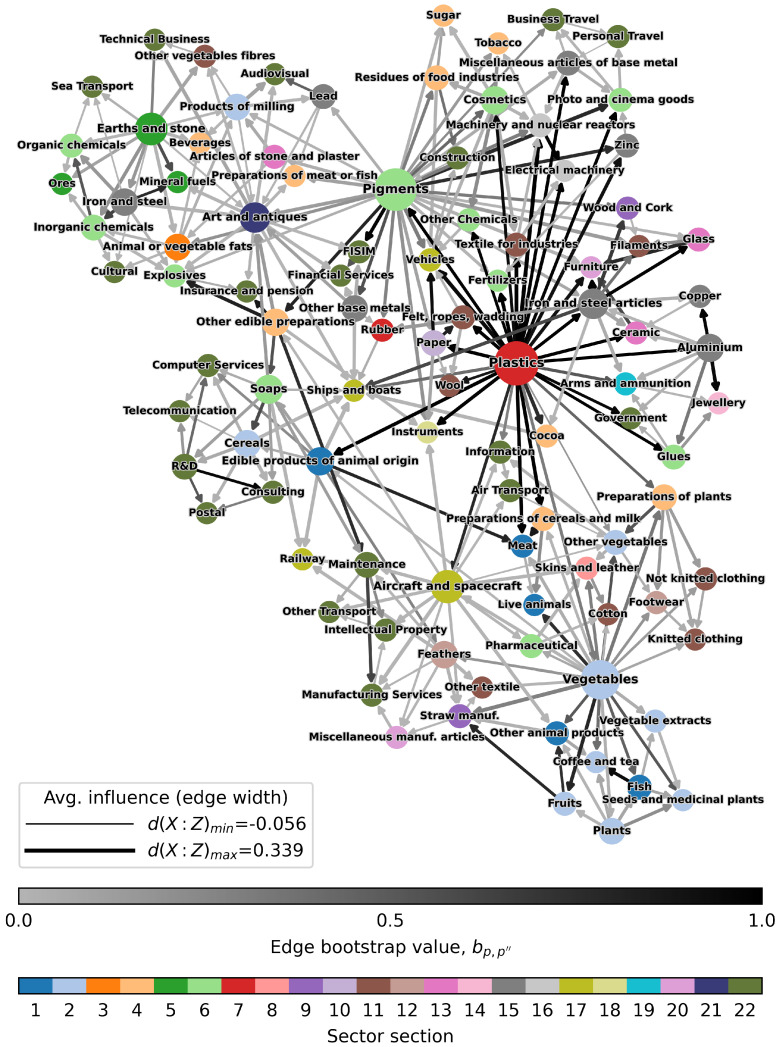
The biPCPG network. The widths of the edges are proportional to the average influence value, $d(p,p^{\prime \prime })$ they represent. The colours of the edges are proportional to their bootstrap value, $b_{p,p^{\prime \prime }}$. The darker the edge, the more reliable it is. Node colours represent the sector section each product and service belong to. Node sizes are proportional to out-degree. The node layout was found using the ForceAtlas2 algorithm [44].

The network *G* resulting from the application of the biPCPG method to our dataset is shown in Figure 3. This network displays some interesting results with a few distinct hub nodes. The most noticeable of these nodes are “Plastics”, “Pigments” and “Vegetables” nodes. Hub nodes in the network also tend to have high average influence on other nodes in the network, this being displayed by the width of the edges stemming out of them. The colour of the edge represents its bootstrap value. We note that the hub nodes are also the source of most of the darker edges in the network, i.e., the most reliable edges, especially the “Plastics” node, whose edges bootstrap values are very high.

The resulting network also displays distinct clusters of intuitively related economic sectors. For example, the most recognisable “food and plant” cluster can be found at the bottom-right of the network, surrounding the “Vegetables” hub node. At the top-left of the network, we can observe another distinct cluster containing several sectors related to chemicals or raw materials. Finally, on the top-right of the network, surrounding the “Plastics” and “Pigments” nodes, one can find a “macro-cluster” formed mostly by industrial and manufacturing sectors.

It is worth noting that, while most edges connect intuitively related sectors, the are several cases of less-intuitive connections spread around the network. This causes the inclusion of some of these seemingly unrelated sectors in some of the clusters mentioned above. This is partially due the original construction of the PCPG algorithm, which ensures a fixed number of edges to be included in the network. Therefore, edges representing small influences among sectors could be forced to be included in the network. In our case, the biPCPG network obtained contains around 5% of edges representing Average influence values of 0.05 or smaller.

### 4.2. Assortativity Analysis

As described in Section 2, the 100 sectors in our dataset can be grouped into 22 groups of sectors called *sections*. Furthermore, a key metric within the field of economic complexity is the *complexity* of a product or service, which measures the capabilities needed by a country to produce it (see Appendix A). In order to better understand the structure of this network, and by extension the information contained in it, one can then investigate its *homophily* or *assortativity* according to these characteristics. Roughly speaking, this is the tendency for nodes belonging to the same group to be connected to each other. In this paper, we make use of two different assortativity metrics which we describe below. The motivation behind this analysis is to assess if our framework generates a meaningful network which is able to synthesise information about the system.

#### 4.2.1. Assortativity by Unordered Characteristics

This quantity is used to measure the assortativity between, for example, nodes with an associated qualitative characteristic such as, in our case, sector sections, *s* (see Section 2). The *assortativity coefficient* is defined as [45]

(11) $$s_{s}\;=\;\frac{TrF-∥F^{2}∥}{1-∥F^{2}∥}$$

where entries of the matrix F are the fractions of edges in the network that connect a vertex of section *s* to one of section $s^{\prime }$, and ||X|| is the sum of all elements of a matrix X [45]. Therefore the numerator is a quantity that measures the fraction of the edges in the network that connect vertices of the same type (i.e., within-section edges) minus the expected value of the same quantity in a network with the same community divisions but random connections between the vertices. The denominator is one minus the same expected value.

This formula gives $s_{s}=0$ when there is no assortative mixing and $s_{s}=1$ when there is perfect assortative mixing. For a perfectly disassortative network, the value is in the range $-1\leq s_{s}<0$ (see [45] for its interpretation). We evaluate this metric for the section of sectors described in Section 2, denoting this by the subscript *s*.

#### 4.2.2. Assortativity by Scalar Characteristics

A measure of assortativity for numeric quantities associated with nodes can also be defined [45]. First, note that the entries of the matrix F are the fraction of all edges in a network that connect nodes with associated scalar values *q* and $q^{\prime }$. Note that the values *q* and $q^{\prime }$ are discrete—in our case these are the *Complexity rank* [17] of sectors—computed by taking average complexity *value* of each product (across the available years in our dataset) and ranking these averages from highest to smallest. The complexity of a product or service is a well-known quantity in the economic complexity literature that describes the capabilities needed by a country to produce it, see Appendix A for its definition. The *numeric assortativity coefficient* is defined as

(12) $$s_{q}\;=\;\frac{\sum_{q,q^{\prime }}qq^{\prime }\left(F_{q,q^{\prime }}-a_{q}b_{q^{\prime }}\right)}{\sigma_{a}\sigma_{b}}$$

where $a_{q}=\sum_{q^{\prime }}F_{q,q^{\prime }}$, $b_{q^{\prime }}=\sum_{q}F_{q,q^{\prime }}$ and $\sigma_{a}$ and $\sigma_{b}$ are the standard deviations of the distributions of $a_{q}$ and $b_{q^{\prime }}$, respectively. The value of $s_{q}$ is in the range $-1\leq s_{q}\leq 1$ with $s_{q}=1$ indicating perfect assortativity and $s_{q}=-1$ indicating perfect disassortativity. Typically, assortativity values in the range 0.3–0.7 are considered to indicate a significant community structure in social networks (higher values are rare) [46,47].

#### 4.2.3. Assortativity Results

The results for the two assortativity metrics defined above are as follows:

- assortativity by sector section = $s_{s}$ = 0.08 (0.15 without FDR correction);
- assortativity by sector mean complexity rank = $s_{q}$ = 0.19 (0.31 without FDR correction).

These results indicate that the structure of the resulting biPCPG network encodes information efficiently. Firstly, the *Assortativity by sector section*, $s_{s}=0.15$, is positive, this means that sectors that belong to the same *section* (see Section 2) tend to be connected in the network, i.e., they influence each other. The section of each sector is reflected in Figure 3 by the colour of the node. The most evident clustering of sectors within the same section is found at the top of the plot where a highly connected cluster of service sectors is found.

Furthermore, the moderately high *Assortativity by sector mean complexity rank*, $s_{q}=0.19$, indicates that sectors around the same level of complexity tend to influence each other. This makes sense intuitively since, according to the economic complexity literature, these tend to be connected in other networks that describe the relationship among products (e.g., product space network, product taxonomy network [21,22]).

### 4.3. Community Detection on the biPCPG Network

We apply a well-known community detection algorithm for directed networks based on spectral optimisation [48]. The modularity, or quality function, to be maximised is

(13) $$Q^{\mathrm{dir}}\;=\;\frac{1}{m}\sum_{p,p^{\prime \prime }}\left(A_{p,p^{\prime \prime }}-\frac{k_{p}^{\mathrm{out}\;}k_{p^{\prime \prime }}^{\mathrm{in}\;}}{m}\right)\delta \left(\nu_{p},\nu_{p^{\prime \prime }}\right)$$

where A is the adjacency matrix, $k_{p}^{\mathrm{in}}$ and $k_{p}^{\mathrm{out}}$ are the weighted in-degree and out-degree of node *p*, *m* is the total edge weight in the network, $\nu_{p}$ is the community of node *p* and $\delta \left(\nu_{p},\nu_{p^{\prime \prime }}\right)\;=\;1$ if $\nu_{p}=\nu_{p^{\prime \prime }}$ and 0 otherwise. This method does not require any parameter choices relating to community size or number of communities; however, adaptations of this method that allow for these choices are available in the literature. It is worth pointing out that, for the analysis carried out in this paper, edge-weights are all set to 1. In Equation (13), this makes the weighted in-degree and out-degree simply the in- and out-degree as well as fixing m=294, the total number of edges in the network.

Since there is no universal definition for communities in directed networks, we also apply the same community detection algorithm for the undirected version of the biPCPG network $G^{\mathrm{und}}$. In this case, the modularity to be maximised is given by

(14) $$Q^{\mathrm{und}}\;=\;\frac{1}{2m}\sum_{p,p^{\prime \prime }}\left(A_{p,p^{\prime \prime }}^{\mathrm{und}}-\frac{k_{p}k_{p^{\prime \prime }}}{2m}\right)\delta \left(\nu_{p},\nu_{p^{\prime \prime }}\right)$$

where $A^{\mathrm{und}}$ is the undirected adjacency matrix which defines the undirected network $G^{\mathrm{und}}$. This can be obtained from the adjacency matrix, A, which defines the directed biPCPG network *G* as follows

(15) $$A_{p,p^{\prime \prime }}^{\mathrm{und}}\;=\;\left\{\begin{array}{cc} 1 & \mathrm{if}\;A_{p,p^{\prime \prime }}\;=\;1\;\mathrm{or}\;A_{p^{\prime \prime },p}=1,\\ 0 & \mathrm{otherwise}. \end{array}\right.$$

This allows us to qualitatively assess if the structure of the biPCPG network is sufficient for reasonable communities to be detected, without the bias of the information contained in the average influence or bootstrap values associated to edges. We implement this algorithm via the *leidenalg* Python package (version 0.8.4) [49], an implementation of the *leiden* algorithm for modularity optimisation.

Note that optimising modularity is an NP-hard problem [50], and therefore heuristics have to be implemented for algorithms to be efficient. One of the steps in the *leiden* algorithm used here involves selecting a random community for a node to be added to. However, this randomness can be controlled via a *seed* to the random number generator. This makes the process deterministic such that the same communities are selected every time the algorithm is run on a given network using the same seed value. In our analysis, we tested several seed values finding that the detected communities varied only for a few nodes, with many seed values returning the exact same partitions. The results shown in Section 4.3 were found using 1 as the seed, as well as for many other seed values tested.

Furthermore, we compare the the communities obtained for the directed and undirected versions of the network for seed values 1, …, 1000 via the *Adjusted Mutual Information* [51]. Take, for example, our set of *P* of *N* sectors and consider two partitions of *P*, namely $U=\{U_{1},U_{2},\ldots ,U_{J}\}$ with *J* pairwise-disjoint clusters found by maximising $Q^{\mathrm{und}}$ for the undirected version of the network, and $V=\{V_{1},V_{2},\ldots ,V_{D}\}$ with *D* pairwise-disjoint clusters found by maximising $Q^{\mathrm{dir}}$ for the directed version of the network. The AMI between the two partitions is then defined as

(16) $$AMI\left(U,V\right)\;=\;\frac{MI(U,V)-E\{MI(U,V)\}}{\max\{H(U),H(V)\}-E\{MI(U,V)\}}$$

where MI(U,V) is the mutual information between two partitions, E{MI(U,V)} is the expected mutual information and H(U) and H(V) are the entropy values associated to partitions *U* and *V* respectively. The AMI equals 1 when two partitions are exactly the same and 0 when the MI between them equals its expected value and therefore serves as a similarity measure for the two partitions, for further details on its calculation see [51]. In Section 4.3, we give the result for the *average* AMI obtained for the 1000 seed values tested using the *scikit-learn 0.23* Python package.

#### Community Detection Results

The community detection procedure described above yielded 5 distinct communities when applied on the undirected biPCPG network, $G^{\mathrm{und}}$, which we denote communities ν=1,…,5. These communities have 31, 22, 21, 13 and 13 sectors contained in each of them, respectively.

**Figure 4:**
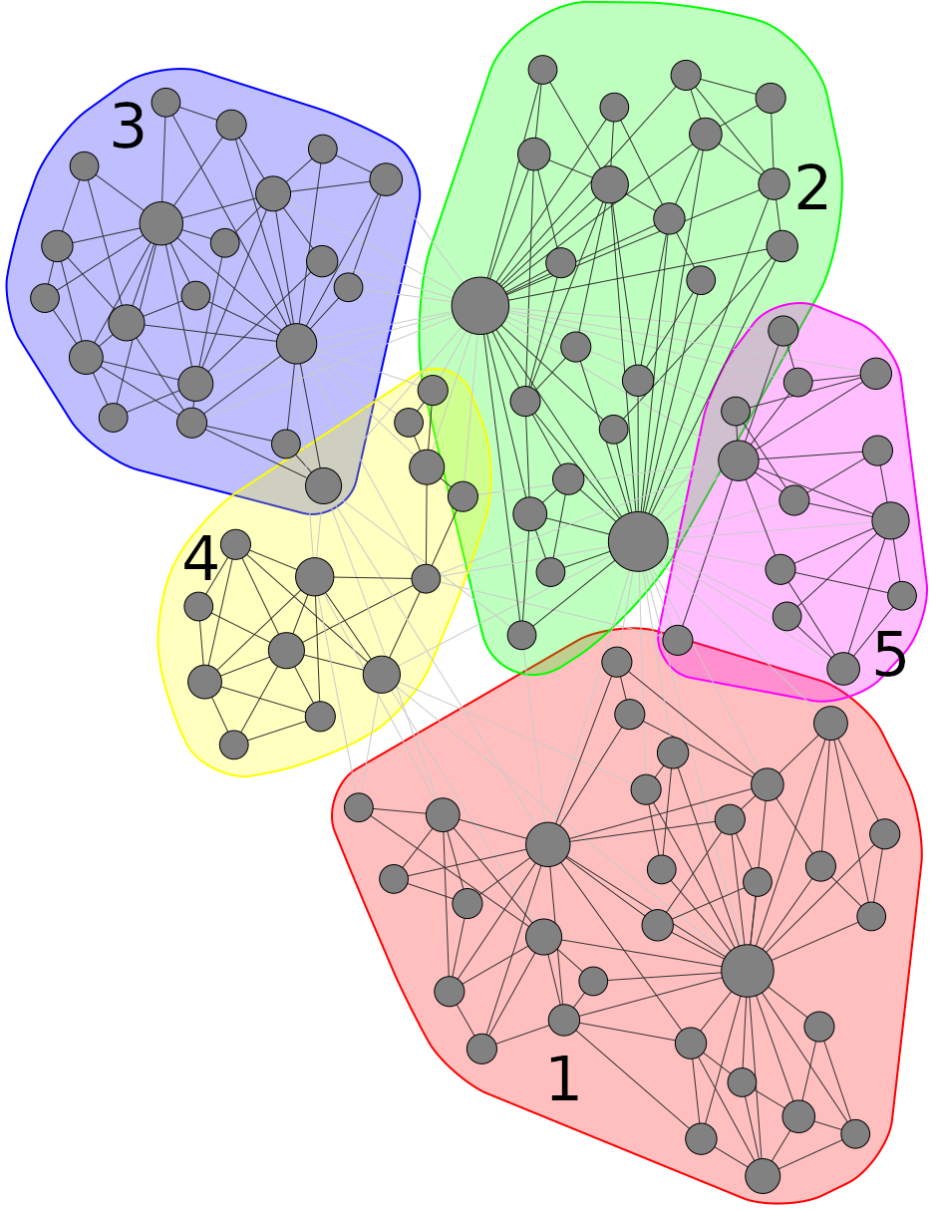
biPCPG network, *G*, resulting from the application of the PCPG algorithm on the mean correlation matrix $\bar{K}$ between sectors’ RCA time series. Nodes are grouped by their community, ν, found by maximising modularity in the network. The node layout was found using the ForceAtlas2 algorithm [44].

The detected communities in the network can be seen highlighted in Figure 4. When comparing with Figure 3, which shows the network highlighting the section of each sector, one can see that the detected communities partition the network into groups that contain intuitively related sectors. For example, communities 2, 3 and 5 contain mostly nodes related to industrial and chemical sectors, while community 1 captures the “food and plant” cluster described above as well as some service sectors. Finally, for community 4, it is slightly more difficult to find a common theme. However, it is worth noting that over half of the sectors it contains are service sectors.

**Figure A2:**
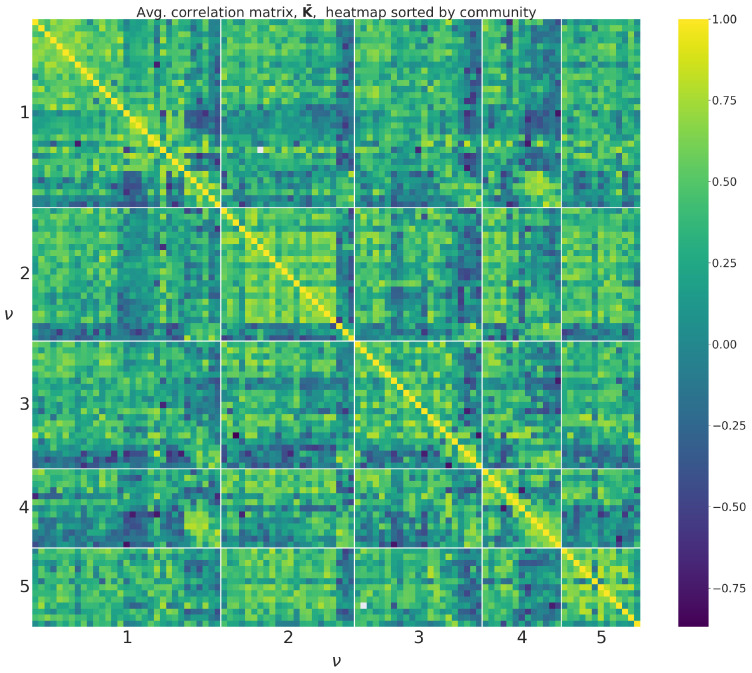
Average correlation matrix $\bar{K}$ sorted by communities ν found by maximising modularity.

**Figure A3:**
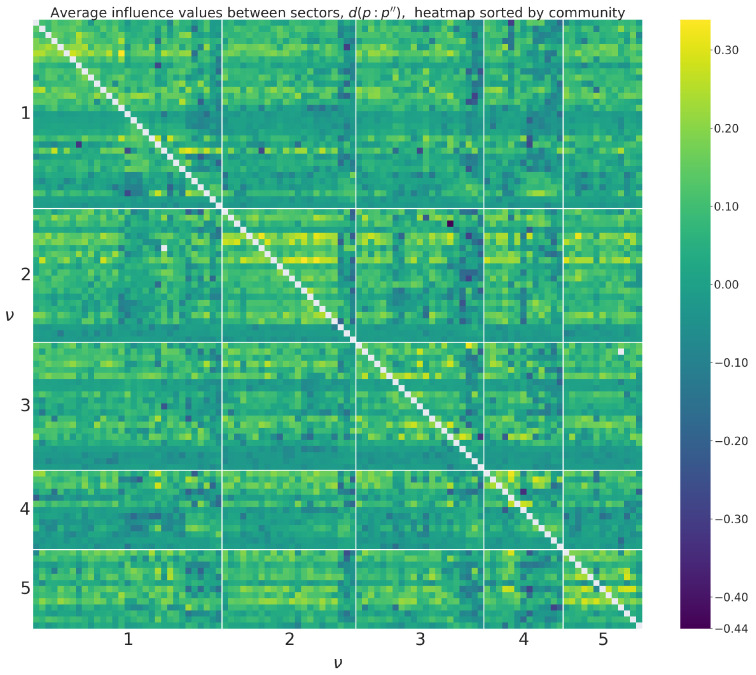
Matrix showing average influence values between products $d(p:p^{\prime \prime })$ sorted by communities ν found by maximising modularity. Entries in white indicate that the average influence of a sector on itself is undefined.

The information structure these communities contain can be seen when sorting rows and columns of the average correlation matrix $\bar{K}$ and average influence matrix by community index as seen in Figure A2 and Figure A3 in Appendix C. We can observe, for example, that brighter colours, meaning higher values, are generally found close the diagonal of the matrices (i.e., among sectors within the same community). This is especially noticeable for communities 1 and 2. We can also identify which rows and columns represent service sectors, as these tend to have a lower correlation and average influence values with non-service sectors (depicted in dark blue) and higher values among themselves.

The average *adjusted mutual information* obtained for the 1000 seed values tested is 0.90. This is a very high value which tells us that, on average, the partitions obtained for the directed and undirected versions of the network were very similar. This suggests that the community detection procedure is weakly dependent on the version of the network (directed vs. undirected) as well as the seed value used.

## 5. Discussion

In this paper we have introduced the biPCPG framework, a generalisation of the PCPG [30] algorithm to datasets with a multi-sample and multi-variable structure that allows a statistical significant and robust analysis, mainly by generating confidence bounds via an adapted bootstrapping procedure. We have then applied this new procedure to a recently introduced dataset that integrates the export of physical goods and services data. The proposed procedure allows the generation of a network of these economic sectors whose links represent the average influence in terms of temporal correlation. This can be seen as an an asymmetric formulation of relatedness [26,52]. The resulting network contains several hub nodes with high degree (namely Plastics, Pigments, Iron and steel articles, Preparations of cereals and milk and Aluminium) as well as distinct clusters of intuitively-related economic sectors (such as a food and plant cluster, a services cluster and manufacturing cluster). We find that, in this network, economic sectors display a relatively high assortativity according to their complexity rank and, to a lesser extent, their category.

## 6. Conclusions

In this work, we have introduced an asymmetric definition for relatedness by extending the PCPG methodology introduced in [30] for its use on bipartite datasets, which we call biPCPG. We apply this approach to a recently introduced dataset containing the exports of countries regarding both manufactured products and intangible services. We show that the biPCPG methodology is able to generate a statistically robust network of economic sectors which captures the underlying influence structure int erms of temporal correlations.

This work can be extended in a number of possible directions. First of all, the biPCPG framework can be applied to any temporal bipartite network, such as those of common use in economic complexity, such as the company-technology [9] or the country-scientific field network [29]. Moreover, the adapted bootstrapping procedure can be used to other network-generating techniques based on correlation-filtering to datasets with a multi-sample and multi-variable structure. These techniques include those based on threshold methods [53], the Minimum Spanning Tree [33] and the aforementioned PMFG [31], as well as more recent techniques based on a null-model approach [54]. This would be possible by replacing the last step in our procedure, the original PCPG algorithm, with the correlation-filtering technique of interest. Finally, it would also be particularly interesting to apply our procedure to datasets with the same structure but longer time series, such as financial datasets containing, for example, asset prices at the different exchanges where they are traded. 

## Figures and Tables

**Table 1:** Examples of values used in the computations of influence $d(p,p^{\prime }:p^{\prime \prime })$.

	Variable & Sector		Corr.	Corr.	Corr.	Partial Corr.	Influence
			${\bar{K}}_{p,p^{\prime }}$	${\bar{K}}_{p,p^{\prime \prime }}$	${\bar{K}}_{p^{\prime },p^{\prime \prime }}$	$\rho (p,p^{\prime }:p^{\prime \prime })$	$d(p,p^{\prime }:p^{\prime \prime })$
Ex. 1	p	Cereals	0.024388	−0.017268	0.028770	0.024899	−0.000511
	p′	Telecommunication					
	p″	Other textile					
Ex. 2	p	Audiovisual	0.283807	0.772049	0.368241	−0.000834	0.284641
	p′	Sea Transport					
	p″	Cultural					
Ex. 3	p	Pigments	0.602575	0.064069	0.040062	0.601727	0.000848
	p′	Aluminium					
	p″	Knitted clothing					
Ex. 4	p	Vehicles	0.025574	0.781281	0.542898	−0.760384	0.785958
	p′	Earths and stone					
	p″	Plastics					

**Figure A1:**
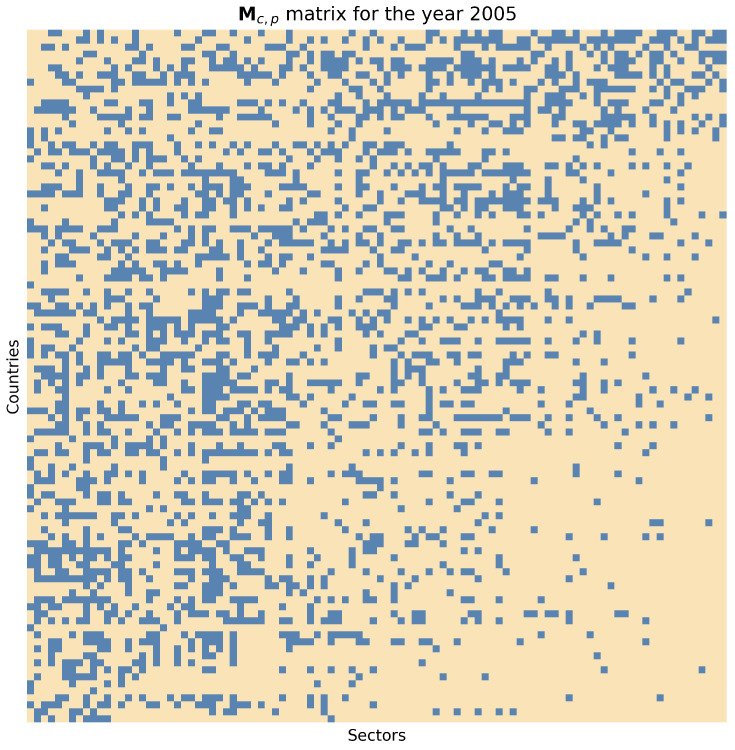
Binary matrix $M^{2005}$ displaying high $RCA_{c,p}$ values for the year 2005. Blue indicates an entry of one and yellow an entry of zero. The triangular structure of the matrix implies a nestedness in the data.

## Data Availability

Raw databases are available from (https://comtrade.un.org, accessed on 13 February 2019) and (https://data.imf.org, accessed on 13 February 2019).

## Abbreviations

The following abbreviations are used in this manuscript:

AMI	Adjusted Mutual Information
BH	Benjamini–Hochberg
biPCPG	Bipartite Partial Correlation Planar Graph
BPM	International Monetary Fund’s Balance of Payments data
EC	Economic Complexity
FDR	False Discovery Rate
HS	Harmonized System
IMF	International Monetary Fund
MST	Minimum Spanning Tree
PCPG	Partial Correlation Planar Graph
PMFG	Planar Maximally Filtered Graph
RCA	Revealed Comparative Advantage
UN-COMTRADE	United Nations Commodity Trade Statistics Database
USD	United States Dollar
WCO	World Customs Organization

## Appendix A. Fitness and Complexity of Economic Sectors

From the matrices containing $RCA_{c,p}$ time series, described in Section 3.3 we can derive the $M^{y}$ matrix which has entries given by

(A1) $$M_{c,p}^{y}\;=\;\left\{\begin{array}{cc} 1 & \mathrm{if}\;RCA_{c,p}^{y}\geq 1,\\ 0 & \mathrm{otherwise} \end{array}\right.$$

where *c* represents a country, *p* represents a product (or service), and *y* represents a given year.

This matrix therefore summarises the countries having a comparative advantage at exporting the different products or services in a given year, or not. Two key quantities from the economic complexity literature are defined using this matrix, namely the *fitness* of countries and the *complexity* of products (or services) [17,55]. The intuition behind these quantities is that the higher the fitness of a country the higher its capability of exporting products of high complexity. It is therefore natural for the fitness to be proportional to the weighted sum of the products of which it is a competitive exporter. The definition of the complexity of a product is more subtle. In general terms, the complexity of a product should be inversely proportional to the number of countries exporting it. We should also note that more economically developed countries tend to have a highly diversified export basket, while less economically developed countries tend to have a much more limited diversification in their exports, and focused on low complexity products. Therefore, the upper bound of a product’s complexity should be determined by the fitness of the countries’ exporting it, with a strong bias towards lower fitness countries: if a product is exported by lower fitness countries, its complexity can not be high. The fitness $F_{c}$ of a country and the complexity $Q_{p}$ of a product (or service) are therefore defined using the following set of coupled iterative equations

(A2) $$\left\{\begin{array}{c} {\tilde{F}}_{c}^{\left(n\right)}\;=\;\sum_{p}M_{cp}Q_{p}^{\left(n-1\right)}\\ {\tilde{Q}}_{p}^{\left(n\right)}\;=\;\frac{1}{\sum_{c}M_{cp}\frac{1}{F_{c}^{\left(n-1\right)}}} \end{array}\right.\to \left\{\begin{array}{c} F_{c}^{\left(n\right)}\;=\;\frac{{\tilde{F}}_{c}^{\left(n\right)}}{{\langle {\tilde{F}}_{c}^{\left(n\right)}\rangle }_{c}}\\ Q_{p}^{\left(n\right)}=\frac{{\tilde{Q}}_{p}^{\left(n\right)}}{{\langle {\tilde{Q}}_{p}^{\left(n\right)}\rangle }_{p}} \end{array}\right.$$

which are iterated until a fixed point is reached [56]. This fixed point has been shown to be stable and not dependent on the initial conditions, which are set to ${\tilde{Q}}_{p}^{\left(0\right)}=1\forall p$ and ${\tilde{F}}_{c}^{\left(0\right)}=1\forall c$ [17]. We use the complexity of products and services in our dataset to calculate an assortativity metric on the network *G* as described in Section 4.2.

It is worth noting that the dataset analysed and similar datasets explored in the economic complexity literature exhibit a nested structure [56]. This nested structure is manifested as a triangular structure in the $M^{y}$ matrices when countries (rows) and sectors (columns) are sorted by their fitness and complexity rank, respectively. This can be seen in Figure A1, which is the $M^{y}$ matrix for the year y=2005.

## Appendix B. Confidence and Prediction Interval Calculations

The 95% confidence interval around a linear fit ${\hat{\mu }}_{y|x_{0}}$ done on *n* data points $(x_{i},y_{i})\;n=1,\ldots ,n$ contains the mean response of new values $\mu_{y|x_{0}}$ at a given value $x_{0}$ with a 95% probability. This is given by

(A3) $$\left|{\hat{\mu }}_{y|x_{0}}-\mu_{y|x_{0}}\right|\;\leq \;T_{n-2}^{.975}\hat{\sigma }\sqrt{\frac{1}{n}+\frac{{\left(x_{0}-\bar{x}\right)}^{2}}{\sum_{i=1}^{n}{\left(x_{i}-\bar{x}\right)}^{2}}}$$

where ${\hat{\mu }}_{y|x_{0}}=a+bx_{0}$ is computed from the linear fit, $T_{n-2}^{.975}$ is the 97.5th percentile of the Student’s t-distribution with n−2 degrees of freedom and $\hat{\sigma }$ is the standard deviation of the residuals in the linear fit given by

(A4) $$\hat{\sigma }\;=\;\sqrt{\sum_{i=1}^{n}\frac{{\left(y_{i}-\hat{y}\right)}^{2}}{n-2}}.$$

The 95% prediction interval around a linear fit ${\hat{y}}_{0}$ is the interval within which a new observation, $y_{0}$, at a given value, $x_{0}$, is found, with 95% probability. This is given by

(A5) $$\left|{\hat{y}}_{0}-y_{0}\right|\;\leq \;T_{n-2}^{.975}\hat{\sigma }\sqrt{1+\frac{1}{n}+\frac{{\left(x_{0}-\bar{x}\right)}^{2}}{\sum_{i=1}^{n}{\left(x_{i}-\bar{x}\right)}^{2}}}$$

where ${\hat{y}}_{0}=a+bx_{0}$ is computed from the linear fit. See [57] for a more detailed description.

## Appendix D. Sector List

**Table A1:** List of product (HS2007) and service (IMF BP6) sector codes in the analysed dataset.

Sector Code	Sector Name	Sector Code	Sector Name
01	Live animals	61	Knitted clothing
02	Meat	62	Not knitted clothing
03	Fish	63	Other textile
04	Edible products of animal origin	64	Footwear
05	Other animal products	67	Feathers
06	Plants	68	Articles of stone and plaster
07	Vegetables	69	Ceramic
08	Fruits	70	Glass
09	Coffee and tea	71	Jewellery
10	Cereals	72	Iron and steel
11	Products of milling	73	Iron and steel articles
12	Seeds and medicinal plants	74	Copper
13	Vegetable extracts	76	Aluminium
14	Other vegetables	78	Lead
15	Animal or vegetable fats	79	Zinc
16	Preparations of meat or fish	81	Other base metals
17	Sugar	83	Miscellaneous articles of base metal
18	Cocoa	84	Machinery and nuclear reactors
19	Preparations of cereals and milk	85	Electrical machinery
20	Preparations of plants	86	Railway
21	Other edible preparations	87	Vehicles
22	Beverages	88	Aircraft and spacecraft
23	Residues of food industries	89	Ships and boats
24	Tobacco	90	Instruments
25	Earths and stone	93	Arms and ammunition
26	Ores	94	Furniture
27	Mineral fuels	96	Miscellaneous manuf. articles
28	Inorganic chemicals	97	Art and antiques
29	Organic chemicals	BXSM_BP6_USD	Manufacturing Services
30	Pharmaceutical	BXSOCN_BP6_USD	Construction
31	Fertilizers	BXSOFIEX_BP6_USD	Financial Services
32	Pigments	XSOFIFISM_BP6_USD	FISIM
33	Cosmetics	BXSOGGS_BP6_USD	Government
34	Soaps	BXSOIN_BP6_USD	Insurance and pension
35	Glues	BXSOOBPM_BP6_USD	Consulting
36	Explosives	BXSOOBRD_BP6_USD	R&D
37	Photo and cinema goods	BXSOOBTT_BP6_USD	Technical Business
38	Other Chemicals	BXSOPCRAU_BP6_USD	Audiovisual
39	Plastics	BXSOPCRO_BP6_USD	Cultural
40	Rubber	BXSORL_BP6_USD	Intellectual Property
41	Skins and leather	BXSOTCMC_BP6_USD	Computer Services
44	Wood and Cork	BXSOTCMM_BP6_USD	Information
46	Straw manuf.	BXSOTCMT_BP6_USD	Telecommunication
47	Paper	BXSR_BP6_USD	Maintenance
51	Wool	BXSTRA_BP6_USD	Air Transport
52	Cotton	BXSTROT_BP6_USD	Other Transport
53	Other vegetables fibres	BXSTRPC_BP6_USD	Postal
54	Filaments	BXSTRS_BP6_USD	Sea Transport
56	Felt, ropes, wadding	BXSTVB_BP6_USD	Business Travel
59	Textile for industries	BXSTVP_BP6_USD	Personal Travel
